# On Treatment of Squint by Operation

**Published:** 1865-03

**Authors:** J. W. Hulke

**Affiliations:** Surgeon to the Royal London Ophthalmic Hospital, Moorfields


### ON TREATMENT OF SQUINT BY OPERATION.

By J. W. HULKE, Esq., Surgeon to the Royal London Ophthalmic Hospital, Moorfields.

[This article is a review of 106 cases of squint treated by operation, with remarks upon the operation. In 50 cases of convergent squint, Mr. Hulke adopted the operation in general use by his colleagues, and which he distinguishes as "the Moorfields' operation." In the remaining 49 he adopted the operation of Von Graefe; which operations he then proceeds to describe.]

In the Moorfields' operation, the eyelids having been separated with a spring speculum, the assistant turns the cornea outwards, seizing, for this purpose, the conjunctiva boldly, close to the outer border of the cornea, with a rats-toothed forceps. The operator seizes, with a similar forceps, a small fold of conjunctiva, nearly midway between the inner edge of the cornea and the caruncle, just over the lower border of the tendon of the Rectus internus, and snips it with slender blunt-pointed scissors, so as to make a small horizontal incision. The fascia, which this incision exposes, is in turn snipt through. Next, a blunt hook, with rather a large curve, is passed through the wound beneath the tendon, which is put on the stretch by slightly raising the hook, and divided with small snips of the scissors between the hook and the sclerotic.

The advantages claimed for this operation are those which it possesses in virtue of its subcutaneous character, and the absence of that recession of the caruncle and undue prominence of the eyeball which are not infrequently observed where the conjunctiva has been freely divided.

I believe that these advantages are gained where care is taken to keep the conjunctival wound small, where unnecessary disturbance of the fascia is avoided by using a hook with not too large a curve, and by not sweeping the hook too widely in search of the tendon, where the tendon is divided strictly at its insertion, and by small snips without a wide separation of the scissorblades, and where the fascia is only cut to the requisite extent for the perfect division of the tendon. Where these precautions are neglected, the Moorfields' operation loses somewhat its subconjunctival character, and should an unnecessary division of the fascia oculi have been made, retraction of the caruncle will hardly be absent when cicatrisation is complete.

The objections that the operation is not so easily performed, and that the complete division of the tendon is not so certainly known as where a free division of the conjunctiva and fascia over the tendon is practised, deserves little consideration; for the greater difficulty, if indeed it exists, is more than outweighed by the superior results of the subconjunctival operation, and because the surgeon may rest assured that the tendon has been thoroughly divided if he can bring his hook up to the edge of the cornea. The thrombus and ecchymosis, which are not very infrequent after this operation, are more real objections, though they may be partly avoided by making a counter-wound over the upper edge of the tendon, under cover of the upper eyelid.

In v. Graefe's operation, the eyelids are separated, and the eye is turned outward by an assistant, in the same manner as in the Moorfields' operation. The surgeon makes a small opening in the conjunctiva, with sharp-pointed scissors (which are curved on the flat, and have an eccentric hinge), between the insertion of the tendon and the inner edge of the cornea, but rather nearer the upper or lower edge of the tendon. He then slides the scissor-points beneath the conjunctiva, and snips a small hole in the fascia, through which he passes a hook (with a slightly swollen end and shorter curve than that used in the Moorfields' operation) under the tendon, which he stretches by pushing it on the convexity of the hook towards the cornea, and cuts at its insertion. The external wound need scarcely be larger than in the Moorfields' method, and when the operation has been well performed it as rarely inflames and suppurates. The button of granulations, which is not infrequently very troublesome after operations where all the tissues have been freely divided, occurred in only one of my 49 cases. I think there is less infiltration than after the Moorfields' method, the directness of the wound allowing the blood to escape readily. In both methods I think that the tendon is cut with equal certainty, and at the same point.

[After giving a tabular view of the cases, the author describes "Pritchett's operation" as follows:]

In this operation, the conjunctiva is divided near the inner border of the cornea, and dissected in a flap, together with the fascia off the inner side of the globe. This liberates the tendon of the Rectus internus from its acquired faulty place of insertion. The Rectus externus is next divided, in order to allow a sufficient inversion of the cornea. Lastly, the wound at the inner side of the eye is closed with fine silk stitches, after removing so much of the free edge of the conjunctival flap, that the closure of the wound draws the sunken caruncle forward, and gives the cornea a slight convergence. The tendon of the Rectus internus which has been brought forward with the fascia and conjunctiva, unites itself to the globe at an anterior point, nearer the situation of its normal insertion. The fine stitches give little trouble; they fall out in a few days, or they may be removed at the end of a week. Slight convergence is always desirable at first, because the cicatrix at the inner side of the eye always yields to some extent, and if at the time of the operation the vertical meridian of the cornea has only been brought to the middle of the lid, the final correction of the divergence will be imperfect.—*Ophthalmic Hospital Reports, Vol.* iv, 1864, *p.* 160.

